# Advances in electrochromic device technology through the exploitation of nanophotonic and nanoplasmonic effects

**DOI:** 10.1515/nanoph-2022-0670

**Published:** 2023-01-26

**Authors:** Eric Hopmann, Wu Zhang, Haizeng Li, Abdulhakem Y. Elezzabi

**Affiliations:** Ultrafast Optics and Nanophotonics Laboratory, Department of Electrical and Computer Engineering, University of Alberta, Edmonton, AB T6G 2V4, Canada; Optics & Thermal Radiation Research Center, Institute of Frontier and Interdisciplinary Science, Shandong University, Qingdao, Shandong 266273, China

**Keywords:** electrochromism, Fabry–Perot, nanophotonics, nanoplasmonics, optical modulation, WO_3_

## Abstract

Research regarding electrochromic (EC) materials, such materials that change their color upon application of an electrochemical stimulus, has been conducted for centuries. However, most recently, increasing efforts have been put into developing novel solutions to utilize these on-off switching materials in advanced nanoplasmonic and nanophotonic devices. Due to the significant change in dielectric properties of oxides such as WO_3_, NiO, Mn_2_O_3_ and conducting polymers like PEDOT:PSS and PANI, EC materials have transcended beyond simple smart window applications and are now found in plasmonic devices for full-color displays and enhanced modulation transmission and photonic devices with ultra-high on-off ratios and sensing abilities. Advancements in nanophotonic ECDs have further decreased EC switching speed by several orders of magnitude, allowing integration in real-time measurement and lab-on-chip applications. The EC nature of such nanoscale devices promises low energy consumption with low operating voltages paired with bistability and long lifetimes. We summarize these novel approaches to EC device design, lay out the current short comings and draw a path forward for future utilization.

## 1 Introduction

The electrochromic (EC) effect has historical significance with its origins reaching far back into the 19th century, when Berzelius and Wöhler found that chemical reduction of yellowish stoichiometric WO_3_ colors it blue [1]. Since then, the intriguing idea of modulating and manipulating light and heat transmission through redox cycling of an electrochromic material has spawned a various of EC devices including smart windows [1–6], electronic skins [7–10], displays [11–16], sensors [17–19] and tunable mirrors [20–22]. Electrochromism bears several promising features that favor device integration, such as low operating voltages (Δ*V* < ±2 V) [23–26] and high electrochemical bistability times [27–29] while simultaneously offering a significant change in the dielectric properties [15, 30, 31]. Due to the significant similarities between EC coloration and electrochemical energy storage, devices based on said effect have since emerged as serious contenders even in energy storage applications in supercapacitors and battery-type platforms [32, 33]. Conventional ECDs show about 80% transmission modulation with switching speeds of several seconds and colors limited to the naturally arising colored and bleached states [33, 34]. However, since the beginning of the 21st century, several groups, with even more approaches have been attempting to unlock the full potential of the dynamic change in dielectric properties of EC materials through exploitation of nanophotonic and nanoplasmonic device architecture. In such a way, resonant and nonresonant photonic effects and strong localization of light are exploited to enhance the EC effect and give it new facets. Hence, advanced EC devices (ECDs) have gained traction by exploiting localized surface plasmon resonances (LSPR) for sensing and colorcoding [35–37], introducing full-color tunability in cavity geometries [31, 38, 39], or exhibiting ultra-high modulation in photonic waveguides [40–42].

With the rapid pace of advanced ECD development, a comprehensive study of these novel platforms is necessary to give an overview of the directions and emphasis this new branch of research is taking on. Herein, we lay out the basic principles for nanoplasmonic and nanophotonic devices with enhanced functionality due to the exploitation of the EC effect. First, we will introduce the basics of ECDs involving the EC effect and some respective key device designs, while at the same time highlight their fundamental functionalities and novelties.

## 2 Electrochromic device architecture

**Figure 1:**
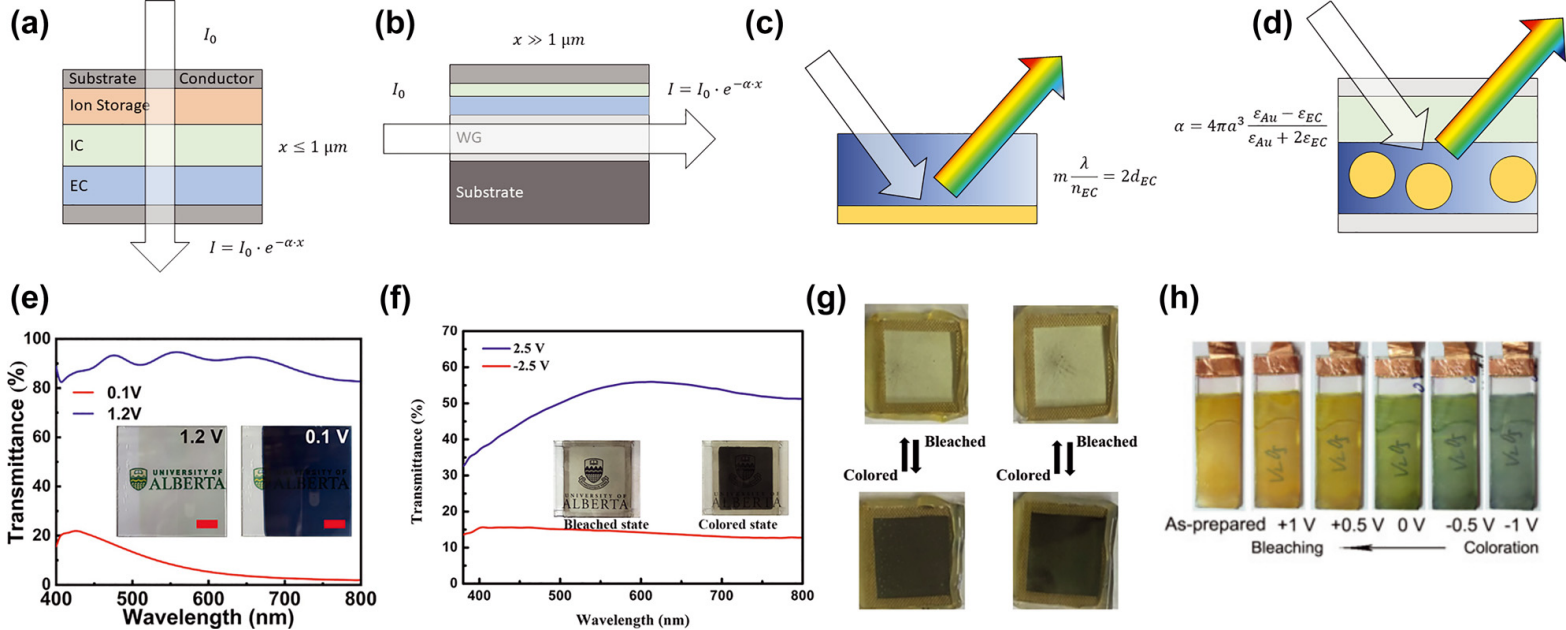
Examples of EC devices and color changes. (a) Conventional ECD design for EC smart windows. (b) Waveguiding architecture with light path perpendicular to ion diffusion. (c) Fabry–Perot style cavity device. (d) Color change device based on plasmonic nanostructures. (e)–(h) Color changes of WO_3_ [44], MoO_3_ hybridized WO_3_ [6], NiO [48], and V_2_O_5_ [49].

Since 1969, when Deb et al. introduced the first of its kind ECD, the basic architecture of an ECD device has remained relatively unchanged. An ECD is comprised of typically five functional parts, including two (transparent) conducting electrodes, an ion storage layer, the electrochromic film, and an ionic conductor (IC) connecting both while providing electrical insulation (Figure 1(a)) [43]. In advanced ECDs this conventional architecture is often changed to enhance absorption or color change through increasing the interaction volume, such as in waveguide applications (Figure 1(b)) or exploit photonic resonances such as Fabry–Perot cavities (Figure 1(c)) and plasmonic scattering (Figure 1(d)). Electrochromic materials in general are materials exhibiting two distinct colors for different oxidation states, such as WO_3_, V_2_O_5_, NiO and Mn_2_O_3_. In such an ECD arrangement, the application of a potential would lead to the intercalation of mobile ionic species from the IC into the EC material. Such ionic species are commonly monovalent ions of the alkali metals lithium (Li^+^) and sodium (Na^+^) or protons (H^+^) but can differ depending on the application and the electrolyte material [44–46]. Upon intercalation of these additional positive charges into the EC material, electrons flow from the electrode to restore the net-zero charge of the EC electrode. In this way, the electrons fill up valence bands, which in the case of WO_3_ leads to the reduction of W^6+^ to W^4+^. The free valence electrons further alter the dielectric properties of the EC material as they introduce free carrier absorption, as well as change the dielectric permittivity of the material. Hence, the color of a WO_3_ film changes from colorless to blue during cathodic reduction (Figure 1(e) and (f)). Other examples of color change are brown to colorless for NiO (Figure 1(g)) oxidation and bluish-green to yellow for V_2_O_5_ (Figure 1(h)). Such a change in color is owed to the different amount in electrons in the EC material before and after ion intercalation takes place. A change in electron density will always change the spectroscopic properties of any given material, but we only refer to materials as electrochromic when one or both of the color states fall into the visible range. Moreover, the intercalation of ions into WO_3_ not only leads to the onset of optical absorption with the extinction coefficient reaching *k* = 0.6, but it also changes the WO_3_ refractive index from roughly *n*_bleached_ = 2.1 to *n*_colored_ = 1.8 [15, 47].

### 2.1 Common performance metrics for ECDs

While the most important metrics in an EC material or device are its color change and contrast, there are several defining factors and terminologies to describe and quantify the operation of EC devices. These performance metrics are by no means standardized but are often comparable throughout the reported publications in this field. In addition to the color change of the material, it is important first before staring the discussion process to define common metrics, such as optical contrast, switching speed, and efficiencies so that the readers can familiarize themselves with the exact definition used for each metric. Nonetheless, to ensure consistency on the reported metrics in the field, a set of standardized definitions are urgently needed [33].

#### 2.1.1 Optical contrast

Typically, the optical contrast of an EC material is defined as the percentage change in optical transmission between the oxidized and reduced state of the material. For this purpose, the absolute change of transmission is measured at either the wavelength of maximum absorbance or often at *λ* = 632 nm for tungsten oxides:

(1) $$\Delta \%T=\%T_{\text{Ox}}-\%T_{\text{Red}}$$

Additionally, advanced ECDs with ultra-high modulation depth, such as those discussed in this review, necessitate the use of the change of transmission in absolute units. The transmission modulation here reaches several orders of magnitude.

(2) $$\Delta T=\frac{T_{\text{Ox}}}{T_{\text{Red}}}$$

This transmission modulation might also be indicated in dB or as the change in optical density (OD).

#### 2.1.2 Switching times

The EC switching time is defined as the time it takes to switch the EC material from one redox state to the other. Hence, the coloration time *τ*_c_ is defined as the time it takes for the material to transition from transparent to absorbent, while *τ*_b_, the bleaching time describes the reverse process. Most commonly, the switching time is calculated at the point at which the EC material achieves 90% of its full switching cycle [33, 44, 50], [51], [52]. Other commonly used values are 75% or 95% [33, 53].

#### 2.1.3 Coloration efficiency

The coloration efficiency (CE) relates the optical contrast of an EC material to the intercalated charge per unit area:

(3) $$CE=\frac{\log\left(T_{\text{Ox}}/T_{\text{Red}}\right)}{Q/A}$$

With *Q*, the intercalated charge in Coulomb and *A*, the area of the charge intercalation in cm^2^. The coloration efficiency is used as a measure for the charge efficiency of the EC coloration effect. Especially in applications such as smart windows, high CE values are beneficial since they infer a superior energy efficiency.

#### 2.1.4 Cycle life

For applications of EC materials in smart windows and photonic devices long-term performance is important. The electrochemical stability is assessed by artificially aging ECDs through repeated redox cycling. Irreversible effects, such as ion trapping, or material detachment, can lead to degradation over time. To date, there is no standard aging time that is commonly used for device testing. Mostly, lifetimes for ECDs are reported in multiples of 1000 redox cycles.

#### 2.1.5 Charge capacity

Charge capacity measurements are dependent on the type of device measured. In capacitor-type ECDs, the charge capacity is either as the areal capacitance (AC) or the specific capacitance (SC), in units of Farads, as the stored charge is dependent on the applied voltage:

(4a) $$AC\left[mF/cm^{2}\right]\;or\;SC\left[F/g\right]$$

Battery-type device performance, on the other hand, is related to the charge stored through the application of a constant current and is, hence, related to mAh:

(4b) $$AC\left[mAh/m^{2}\right]\;or\;SC\left[mAh/g\right]$$

#### 2.1.6 Reversibility

Intercalation and deintercalation into an EC material occur through ionic current flows. The transferred charge, however, is not necessarily the same for both directions. An unbalanced redox process indicates irreversible intercalation of ionic species into the host material or detachment of the EC material into solution. As a measure of reversibility, the anodic and cathodic charge can be compared and related to each other as:

(5) $$\Gamma =\frac{Q_{\text{int}}}{Q_{\text{deint}}}$$

Here, *Q*_int_ and *Q*_deint_ relate to the transferred charge during intercalation and deintercalation as obtained through cyclic voltammetry (CV). The charge values are obtained via integration of the anodic and cathodic part of the CV curve over time.

#### 2.1.7 CIE color code

A major driving force for the investigation of novel and advanced EC devices is the ability to create dynamic full-color displays with such a technology. Unfortunately, color is a rather difficult metric to compare, which is why the CIE color space is important to be able to evaluate the modulation of EC color displays. The CIE color space relates values *X*, *Y*, *Z* to the spectral sensitivity of human eye cone cells. While *L*, *M*, *S* are the three kinds of cone cells with sensitivities in the short (*S*: 420 nm–440 nm), middle (*M*: 530 nm–540 nm) and long (*L*: 560 nm–580 nm) wavelength range. Obviously, these curves present averages, since all eyes’ sensitivity is different. Nonetheless, with the luminance *Y*, *X* a mix of *L*, *M* and *S* and *Z* representing solely short wavelengths, the CIE color space is represented as:

(6) $$\left[\begin{array}{c} X\\ Y\\ Z \end{array}\right]=\left[\begin{array}{ccc} 1.91020 & -1.11212 & 0.20191\\ 0.37095 & 0.62905 & 0\\ 0 & 0 & 1 \end{array}\right]\left[\begin{array}{c} L\\ M\\ S \end{array}\right]$$

### 2.2 Nanoplasmonic electrochromic devices

The appeal of combining electrochromic functionality with plasmonic properties derives from the inherently strong localization of the electric field through the coupling of the incident photon to the free electrons of a metallic structure. Such a localization is utilized in miniaturization of photonic devices, to enhance light matter interaction in transmittance-type devices or for sensing applications. Plasmonic devices and nanostructures have long been investigated for color printing and display applications [54–57]. However, the resonance wavelength of the excited plasmon is fixed by the dimensions of the plasmonic structure and the materials involved. The active control over the refractive index of WO_3_ and other EC materials can change the dielectric environment of such plasmonic devices and structures to achieve a dynamic modulation effect. As an example, the plasmonic resonance of a nanoparticle is introduced in the following. For plasmonic nanoparticles the localized surface plasmon resonance (LSPR) is directly dependent on the density of free surface electrons. Simplified through the Drude model, the free electron density *N* is related to the dielectric environment as follows:

(7) $$\varepsilon \left(\omega \right)=\varepsilon_{\infty }-\frac{\omega_{\text{P}}^{2}}{\omega^{2}+\text{i}\gamma \omega }$$

With $\varepsilon \left(\omega \right)$ the complex dielectric permittivity, *ω* the frequency of incident radiation, *γ* a damping constant of the electron oscillation, *ɛ*_∞_ the dielectric constant of the bulk material and *ω*_P_ the plasma frequency, which is directly proportional to the free electron density *N*:

(8) $$\omega_{\text{P}}={\left(\frac{Ne^{2}}{m\varepsilon_{0}}\right)}^{1/2}$$

Here, *e* is the elemental charge of an electron and *m* its effective mass. *ɛ*_0_ is the dielectric permittivity of free space. For a spherical plasmonic nanostructure, the static polarizability can be formulated as:

(9) $$\alpha_{\text{Sph}}=4\pi a^{3}\frac{\varepsilon \left(\omega \right)-\varepsilon_{\text{med}}}{\varepsilon \left(\omega \right)+2\varepsilon_{\text{med}}}$$

With *a* the particle radius and *ɛ*_med_ the permittivity of the surrounding medium. Resonance is achieved when *α*_Sph_ diverges (i.e., when the permittivity of the metal sphere, $\varepsilon \left(\omega \right)$ , equals −2*ɛ*_med_, the permittivity of the dielectric medium). Hence, the properties and features of the LSPR can be directly influenced by its dielectric environment. Such a change can be induced through EC materials and exploited for dynamic color change of the LSPR in measurement and display applications. Similar derivations can be made for surface plasmon polaritons and gap plasmons, which can be found in literature [58–61].

#### 2.2.1 Active nanoplasmonic arrays

**Figure 2:**
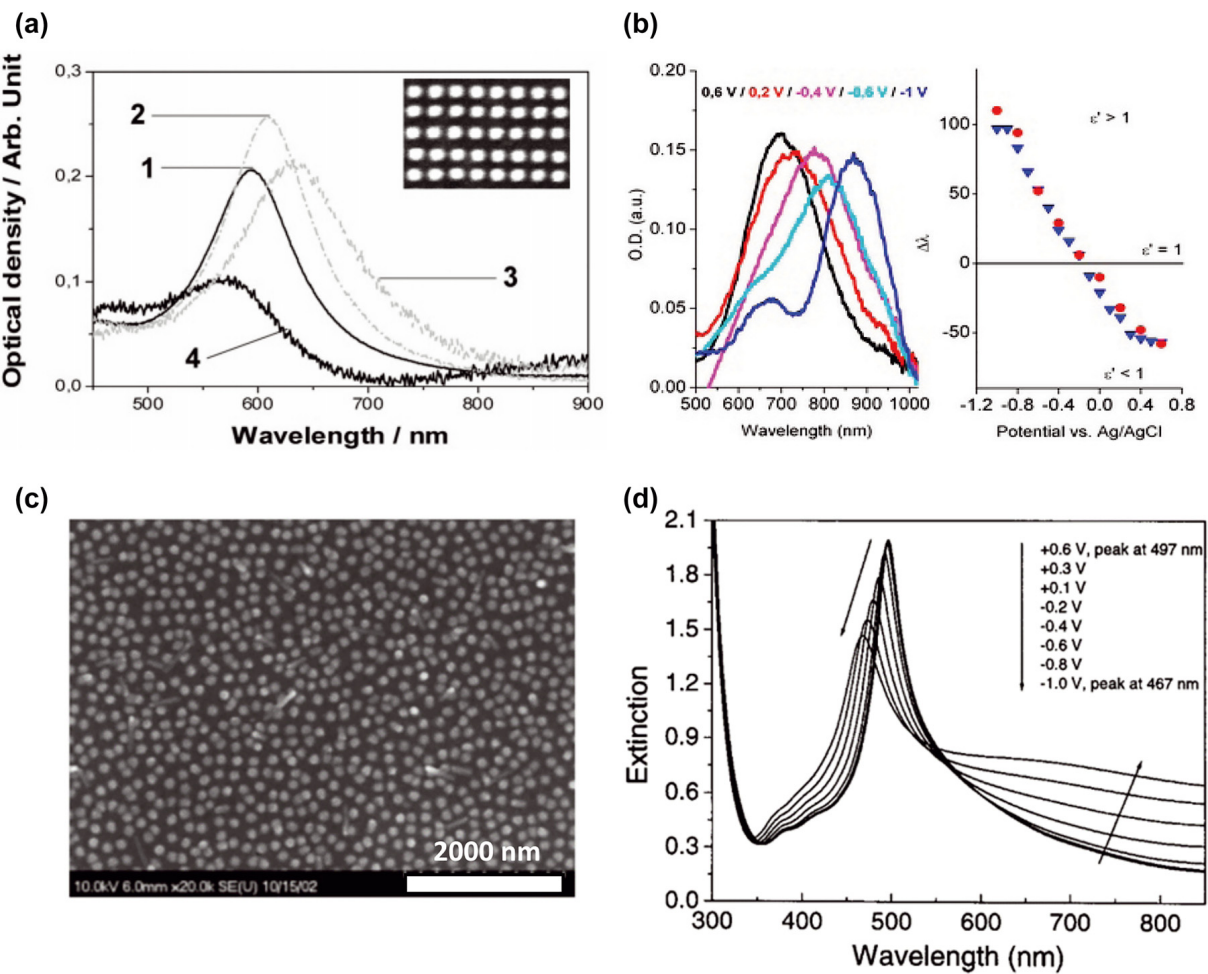
Performance of active gold nanostructure arrays coated with conductive polymers. (a) LSPR of PANI coated Au nanostructures in 1 air, 2 water, 3 PANI reduced, and 4 PANI oxidized. (b) Plasmonic extinction and wavelength shift for PEDOT coated Au array as a function of applied voltage [36, 37]. (c) and (d) Sol-gel modified Ag nanoparticles with a WO_3_ modulation layer. (c) SEM image of the colloidal silver particles. (d) Voltage-dependent extinction spectra for the modified Ag nanoparticle samples [63].

The first mention of polymer coated plasmonic nanostructures for active plasmonic devices was reported in 2005 by Leroux et al. [37]. Plasmonic nanodisk arrays having 150 nm diameter and 40 nm height in several different periodicities, were fabricated through electron beam lithography, with a plasmon resonance in air at *λ*_LSP_ = 597 nm for Δ_*x*_ = Δ_*y*_ = 220 nm grating period. When coated with polyaniline (PANI), the resonance shifts to *λ*_LSP_ = 633 nm due to the increased dielectric permittivity of the environment (Figure 2(a)). During redox cycling of the PANI coating, the resonance shifts to *λ*_LSP_ = 571 nm in less than *t* < 10 s. Furthermore, quenching of LSPR was observed for asymmetrical grating periods when the grid is excited with a polarization along the shorter dimension. Based on a similar Au nanoparticle array, Leroux et al. were able to show not only up to Δ*λ* = 32 nm wavelength shift via redox cycling of the PANI coating, but also a simultaneous increase in optical density of the device from OD = 0.08 to OD = 0.34 [62]. Such a multifunctional EC plasmonic device could serve as an optical switch for color and transmission modulation. In a similar approach based on lithography, Stockhausen et al. employed poly(3,4-ethylenedioxythiophene) (PEDOT) as the conductive polymer modulation layer [36]. Due to the higher change in dielectric properties of the real part of the permittivity at values below *ε*′ < 1, the change in resonance wavelength reaches up to Δ*λ* = 180 nm from approximately *λ*_LSP_ = 720 nm to *λ*_LSP_ = 900 nm (Figure 2(b)). Notably, this study found that Δ*λ* increases when *λ*_LSP_ goes to higher wavelengths. In such devices the wavelength shift is measured in reflection.

Wang et al. were the first to report WO_3_ sol-gel modified plasmonic Ag nanoparticle arrays exhibiting a strong shift in the wavelength of maximum extinction coefficient upon EC cycling of the electrochromic material [63]. The colloidal Ag nanoparticles, as well as the sol-gel modification process, promise ease of fabrication and higher scalability. Figure 2(c) presents an SEM image of the investigated Ag nanoparticles having an average diameter of 100 nm. Correspondingly, a plasmonic extinction resonance at 431 nm is observed when a dip of EC material is casted on indium tin oxide (ITO).

As depicted in Figure 2(d), when coated with the WO_3_ solution, the plasmonic resonance shifts to 498 nm wavelength. Cycling of the plasmonic nanoparticle array in 1 M LiClO_4_ in propylene carbonate (PC), leads to a decrease in the maximum extinction from *k* = 1.95 for the fully oxidized/bleached WO_3_ film to *k* = 1.5 for full reduction of the film. Simultaneously, the plasmonic resonance of the array shifts from 497 nm wavelength to 467 nm. Using polyaniline (PANI)-coated Au nanorods having an average diameter, *d* = 52 ± 3 nm, and an average length, *l* = 111 ± 6 nm, Jiang et al. showed strong LSPR shifts through EC cycling. The coating of PANI shell on the individual Au nanorods was realized through surfactant-assisted chemical oxidative polymerization [64]. The polymerization process allows direct control over shell thickness and, thus, alters the EC properties of the plasmonic structure. The introduced nanorods exhibit wavelength shifts of Δ*λ* = 100 nm with resonance depth amplitude modulation of up to 10 dB. Finite difference time domain (FDTD) simulations of the nanorod LSPR modulation suggest that wavelength modulation of up to Δ*λ* = 130 nm is feasible for such plasmonic nanorod array. Notably, such high dynamic light transmission modulation is achieved using PANI shell thickness *d*_*t*_ < 10 nm. Here, switching speeds around *τ*_c,b_ = 60 s are realized. This colloidal Au nanorod technology provides strong polarization dependence of the light scattered intensity [65]. Under the application of an alternating field at a frequency *f* = 1 Hz, wavelength resonance switching occurs in less than 200 ms for PANI coated Au nanorods. Further investigation of the voltage-dependent behavior of Au nanorods in a custom measurement cell showed a shift in the resonance of PANI-coated Au nanorods by Δ*λ* = 11 nm at an applied DC electric field of *E* = 10 V/µm. A similar wavelength shift was observed for Barium Titanate (BTO) coatings, which indicates that nonlinear optical properties can be utilized in these kinds of devices as well. To overcome the limitations of Au nanorods and particles whose LSPR falls into the red and NIR spectral region, PANI-coated Au nanocubes are employed [66]. These colloidal structures are grown in solution and can be spray coated onto different substrates. The PANI shell helps mitigate the detrimental effect of Au nanocube clustering and introduces an LSPR shift of up to Δ*λ* = 30 nm in the green spectral region.

While colloidal approaches to LSPR tuning show tremendous potential for sensor applications and potential control, the red and the NIR wavelength of nanorod and nanoparticle LSPRs necessitate more complex device design to cover the whole spectral region. Active EC light control over static, plasmonic devices has since been employed in various device architectures. Xu et al. employed the EC polymer PolyProDot-Me_2_ as a fast on-off switching layer for static plasmonic color arrays, achieving less than *τ*_c,b_ = 25 ms switching time for 80% light transmission modulation [67]. This application, however, makes solely the use of the change in extinction that is inherent during redox cycling of the material.

**Figure 3:**
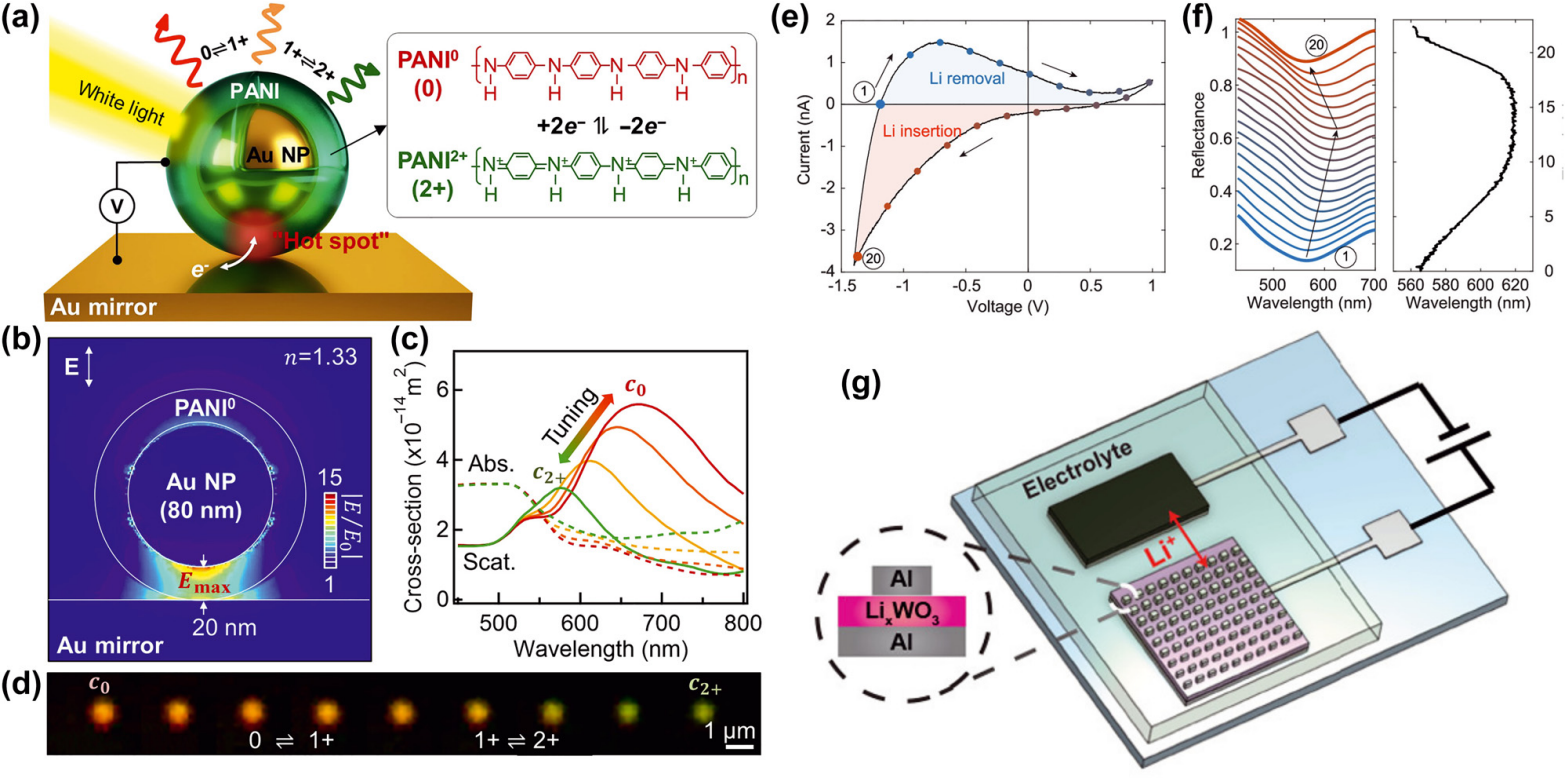
Nanoplasmonic electrochromic color devices for full color modulation. (a)–(d) Plasmonic nanopixels based on a PANI modulation layer. (a) Schematic of the proposed device. An 80 nm Au nanoparticle is coated in a 20 nm-thick PANI shell and placed on a gold mirror, creating a plasmonic “hot spot” between the Au components. (b) FDTD simulation of the electric field distribution and (c) absorption and scattering spectra determined through the numerical approach. (d) Dark-field images of the nanopixel during electrochemical redox cycling [30]. (e)–(g) Dynamic gap plasmon color printing device. (g) Schematic of the EC plasmonic nanodevice with Al nanostructures and a solid PEO electrolyte. (e) CV curve of the plasmonic nanodevice and (f) reflectance spectra (left) and wavelength of minimum reflection (right) taken during cyclic voltammetry, showing a continuous shift [16].

In a colloidal approach to color printing with plasmonic nanostructures, Au nanoparticles are coated with EC PANI [30]. During oxidation of PANI, the plasmonic resonance of the 80 nm diameter Au nanoparticles on an Au mirror, shifts from the red color to yellowish green color (Figure 3(a)). The high spectral bandwidth of the EC color change is owing its presence to the electromagnetic “hot spot” created between the plasmonic nanoparticle and the Au mirror surface. As shown in Figure 3(b), the simulated field enhancement between the Au structures exceeds one order of magnitude. Furthermore, FDTD simulations predict a shift of the reflectance spectra based on the scattering cross-section from *λ*_delith_ = 680 nm to *λ*_lith_ = 590 nm. Owing to the thin PANI shell, extinction losses are low. The experimentally captured dark-field images of the nanoplasmonic pixel device show a clear color change from red to green, having good homogeneity over an area of 1 µm^2^ (Figure 3(d)). Moreover, the plasmonic nanopixel device was shown to exhibit a video frequency (25 Hz) switching with electrochemical stability lasting for more than 3 months. Along the same line, Lu et al. introduced PANI-coated Au nanorods, where they extended the available colors for colloidal plasmonic displays into the blue spectral range, while at the same time achieving switching times as low as 5 ms [68].

Li et al. introduced a plasmonic device consisting of a WO_3_ modulation layer which allows for color switching and resonance modulation through EC cycling (Figure 3(e)–(g)). The device incorporates Al electron beam lithography fabricated rectangular nano blocks having structure sizes ranging between 90 nm and 70 nm in lengths and 50 nm–30 nm in widths with a constant height of 30 nm. The solid polymer ion conductor consists of LiClO_4_ dissolved in polyethylene oxide (PEO), while Li_0.7_FePO_4_ acts as a source and sink for the Li^+^ ions. The device exhibits a high dynamic wavelength shift range between *λ*_delith_ = 620 nm and *λ*_lith_ = 565 nm with up to *r* = 4.1 nm/s modulation speed for the redshift and *r* = 1.8 nm/s for the blue shift (Figure 3(g)). Moreover, the rectangular design shape of the plasmonic scatterers leads to a strong dependence of the reflected spectrum on the polarization of the excitation source. Hence, for white light polarized along the long edge of the rectangle (i.e., along the length (*L*) side), the samples appear blueish in color, while they reflect an orange color when excited with a perpendicular light polarization. The EC nature of the modulation layer allows operation of this device between *V* = −2 V for full lithiation and *V* = +1 V for delithiation, as such creating a low working voltage window. Interestingly, once the external bias is turned off, the bistable nature of the EC effect allows the device to retain its state for tens of minutes.

To increase scalability of nanofabricated modulation devices, Zhang et al. introduced plasmonic resonance shifts in Au nanodisks fabricated through an anodic aluminum oxide (AAO) mask. This facile and scalable process leads to nanodisks exhibiting a plasmonic resonance at a wavelength of *λ* = 550 nm [69]. After coating the nanodisk sample with PEDOT:PSS, the resonance shifts to *λ*_ox_ = 653 nm in the oxidized state and *λ*_red_ = 718 nm in the reduced state. Meanwhile, the reflectance reduces by only 5% from 53% to 48% during electrochemical cycling between Δ*V* = ±1 V. Furthermore, FDTD simulations show the shift in the plasmonic resonance caused by the oxidation of the PEDOT:PSS could accurately describe the wavelength shift and amplitude modulation of the LSPR. For a thin PEDOT:PSS layer of thickness of 27 nm, 95% color modulation could be achieved in less than *t* = 10 s.

There are two types of active plasmonic color and sensing devices investigated for exploitation of the EC effect. On the one hand, scalable approaches on the basis of colloidal nanostructures, which provide sharp plasmonic resonances in the green and red spectral regions. This approach has been realized for Ag and Au plasmonic structures with WO_3_, PANI, and PEDOT as the EC materials. Through different particle dimensions and types, the plasmonic resonance can further be adjusted over a wide range throughout the visible spectrum. Gap plasmon and lithographical approaches based on arrays of nanostructures give an additional degree of freedom due to array periodicities. Certainly, implementation of solid electrolytes is simpler to achieve since the employed structures provide higher mechanical and chemical stability. Notwithstanding, Au-based plasmonic nanostructures are limited to the green and red spectral region, while Ag nanoparticles have shown resonances around 400 nm but lack the electrochemical stability [70]. Hence, a method to overcome these limitations of color and stability is required to pave the for integrated plasmonic display devices. The comparison of some of the reviewed technologies can be found in Table 1.

**Table 1:** Comparison between selected active EC plasmonic modulation schemes. Values for peak wavelength, wavelength shift, and switching times are extracted from figures and graphs from the corresponding references.

Material	Approach	Peak wavelength (nm)	Wavelength shift Δλ (nm)	Switching time (s)	Reference
Au/PANI	Lithography disk array	631	−62	<10	[37]
Au/PEDOT	Lithography disk array	720	+180	/	[36]
Ag/WO_3_	Colloidal sol	498	−31	<5	[63]
Au/PANI	Colloidal nanorods	740	−100	<240	[64]
Al/WO_3_	Lithography rectangle array	620	−55	<20	[16]
Au/PANI	Colloidal spheres with mirror	680	−90	<0.05	[30]

#### 2.2.2 Reversible metal deposition

**Figure 4:**
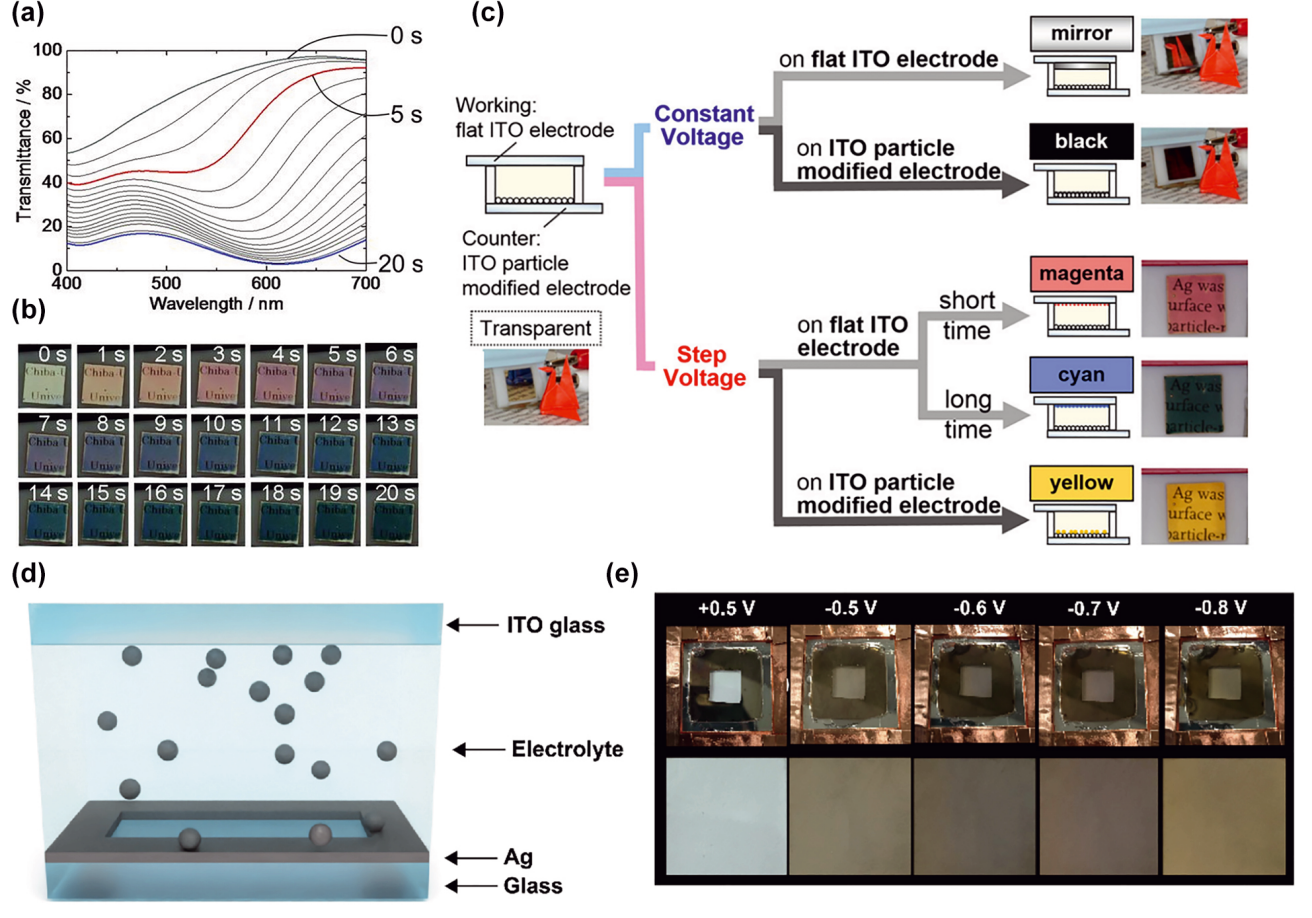
Reversible electrodeposition of metallic nanoparticles. (a) Transmittance spectra of multi-color ECD based on Ag nanoparticle LSPR. (b) Images of the device investigated in (a) [71]. (c) Schematic of the appended ECD presented in (a) and (b) with the addition of the yellow state. Colors and operation modes are strongly dependent on electrode and voltage applied [74]. (d) Schematic of plasmonic Ag ECD based on underpotential deposition of Ag adatoms. (e) Corresponding images of the colors induced by reversible Ag deposition [75].

In addition to these patterning and templating approaches utilizing colloidal nanostructures, there are also some exciting accounts of using reversible metal depositions to generate plasmochromic nanostructures [71–73]. In 2013, Tsuboi et al. introduced an EC mirror device that exhibits multiple color states due to the tuning of the LSPR of reversibly deposited Ag nanoparticles (Figure 4(a) and (b)). Based on the nanoparticular structure of the ITO electrode, the ECD can act as a blackout window or mirror when the Ag is deposited on the flat ITO surface or display the colors red and blue when deposited on an electrode modified to facilitate particle growth. In the case of the displayed colors, the Ag particle size increases from <40 nm for the red resonance to 50 nm for the blue resonance. However, such a device is dynamic, meaning that the blue state is achieved after the red state when the applied potential is held longer (Figure 4(c)) [62]. The introduced device was later improved to further display a yellow state based on the deposition mode and time (Figure 4(c)) [74]. Such a plasmonic ECD provides enhanced tunability of Ag nanostructures and hence LSPR frequency. The plasmonic-based metal deposition approaches are particularly interesting because they not only tune the nanostructures of deposited metal layer, but also display significantly high optical contrast and multiple colors.

In particular, a recent study by Zhang et al. demonstrated a multicolor plasmonic ECD based on the reversible Ag deposition mechanism [75]. The size-tunable Ag nanoparticles can be grown onto a conducting ITO coated glass substrate at an operating voltage as low as −0.5 V and can be efficiently stripped at +0.5 V (Figure 4(d). Furthermore, the ITO electrode can display various plasmonic colors resulting from the resonant interactions between light and the Ag nanostructures which can be manipulated via voltage-induced size and shape control (Figure 4(e)). Here, as the applied negative voltage increases from −0.5 V to −0.8 V, a blueshift of the resonance wavelength of the extinction from 535 nm to 460 nm (i.e., Δ*λ* = 75 nm) can be observed. Upon reversing of the voltage to +0.5 V, dissolution of Ag nanoparticles occurs, and the resonance shifts back to its initial wavelength of 535 nm. Interestingly, the same device can be utilized as a reflective-type ECD with an Ag mirror as the deposition electrode resulting in broad reflectance modulation with peaks ranging from 500 nm to 600 nm. The advantage of the underpotential approach is that no modification of the ITO electrode is necessary and operating voltages stay low.

In line with previous studies on plasmonic-based metal deposition, there is another electrochromic approach enabled by reversible metal deposition [76, 77]. In this approach, thin films of metals are deposited onto the electrode, thus, blocking the light transmission from the device. Even though this reversible metal deposition approach does not lead to a plasmonic color change, devices based on this approach show high optical transmission changes. McGehee et al. demonstrated a dynamic window platform by electrochemically depositing Cu–Pb particles [78]. Using an aqueous electrolyte solution containing Cu^2+^ and Pb^2+^, the authors employed *in situ* cathodically charge to reduce the metal ions in the electrolyte and form a thin-film coating onto the Pt-modified ITO glass. Upon application of −0.6 V, metal deposition on the working electrode results in a high optical transmission contrast of ∼61% within the first 30 s and ∼76% after 180 s. By reserving the voltage polarity and increasing it to +0.8 V, the window’s optical transparency is restored to its original transparency of 81% within ∼70 s. More recently, a polymer-inhibitors-enabled dynamic window via electrochemical deposition of Cu–Bi particles was reported by Strand et al. [79]. By employing polyvinyl alcohol, the window’s color can uniformly tinted over the visible spectral wavelengths (400 nm–900 nm) across the entire window size (>900 cm^2^). Visible transmittance as low as 0.001% and infrared wavelength reflectance of >70% were demonstrated to switch in times of <3 min.

While reversible metal deposition in electrochemical cells has proven to facilitate the development of dynamic plasmonic color devices, the complex nature of these platforms in combination with the reliance on Ag nanostructures has been the limiting for device integration thus far. Color displays incorporating other plasmonic nanostructures, such as Au, Pt, and Cu could provide flexibility and increase the dynamic range of such devices. For applications in display-type devices, the relatively fast switching times >10 s need to be drastically decreased.

### 2.3 Active Fabry–Perot nanocavity devices

Fabry–Perot (F–P) resonators and nanocavities provide structural light colors based on the thickness and refractive index of the dielectric that fills the cavity. For a light at a wavelength *λ*_0_ impinging at normal incidence on an F–P cavity, the resonance condition for a 2D thin-film cavity of thickness t and having areal refractive index of *n*_eff_, is given by:

(10) $$m\cdot \frac{\lambda_{0}}{n_{\text{eff}}}=2d$$

Here, *λ* is the light wavelength, and m being an integer. In 2005, Kammler et al. were first to introduce the concept of utilizing EC materials in a cavity for active optics and photonics [80]. In this pioneering work, solid-state ECDs consisting of WO_3_ as the working electrode, Ta_2_O_5_ as the ion conductor, and NiO as the ion storage layer, are used in a 1D F–P planar cavity to demonstrate minimal, but novel light transmission wavelength modulation by Δ*λ* = 2 nm [31]. Surprisingly, this technology was not investigated further until 11 years later. Starting in 2016, static resonator reflections were used for EC-modulated color imaging and printing. To create narrower resonance and higher chromaticity for cavity-based color printing devices, several works introduced a superstructure, consisting of a plasmonic nanohole array (NHA) as the top mirror of the cavity [81–83]. It was shown that the nanohole arrays create plasmonic resonances that spectrally overlap with the cavity reflection and therefore, act like spectral filters for the cavity. However, such devices lack dynamic response and active tuning, but almost certainly inspired works on EC NHA cavities in the years to follow.

**Figure 5:**
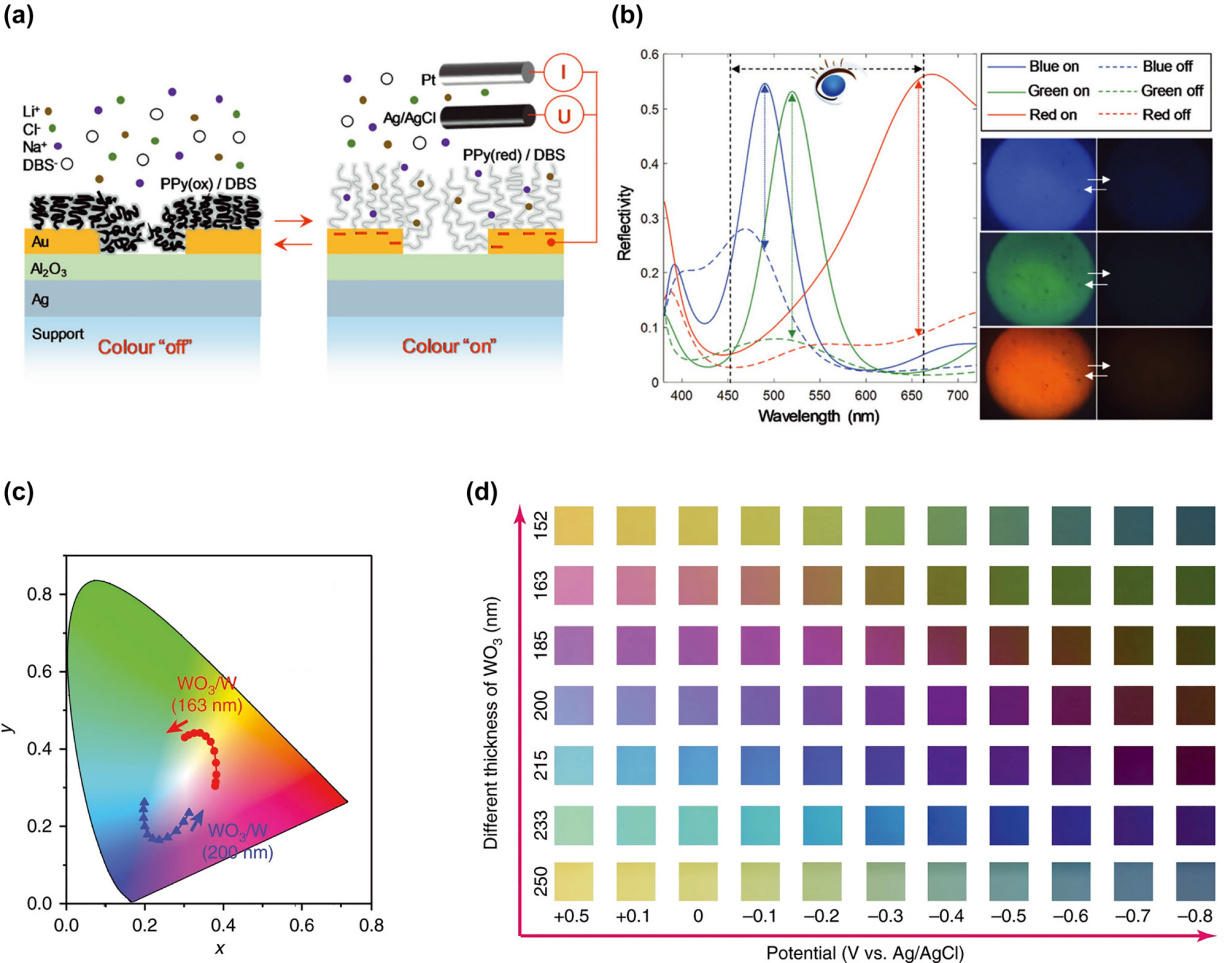
EC switching of MIN resonant cavity. (a) Schematic of the active MIN cavity with an Au NHA and polypyrrole as the EC modulation material. (b) Contrast induced through EC cycling of PPy under a viewing angle between 0° and 14° [84]. WO_3_-based resonant nanocavities for color generation. (c) CIE color space for WO_3_/W nanocavities as a function of WO_3_ layer thickness and intercalation. (d) Dynamic reflectance of different thickness WO_3_/W cavities as a function of applied voltage in a 1 M LiClO_4_ electrolyte [39].

To overcome the static nature of these metal-insulator-nanohole array (MIN) structures, Xiong et al. introduced several device design configurations utilizing conjugated EC polymers for on-off switching. The reflection of the cavity is first determined through the thickness of a dielectric Al_2_O_3_ layer placed between an Ag mirror and an Au NHA. By adjusting the period of the array, the plasmonic NHA is designed to have a spectral overlap with the F–P resonance [81, 84]. The plasmonic NHA exhibited insignificant change in the reflectivity for viewing angles between 0° and 45°. For EC-based on–off switching, polypyrrole (PP) is first electropolymerized onto the Au NHA (Figure 5(a)). The PP layer is then oxidized or reduced by applying a voltage between the NHA and a Pt counter electrode. In its oxidized state, PP is highly lossy, leading to the strong optical modulation over 25% in the blue region and up to 50% in the red spectral region (Figure 5(b)). The highly chromatic blue, green and red color emitting cavities can be completely turned off. Not only does such a device exhibit fast switching times in the range of several 10 ms, it also consumes less than 0.5 mW/cm^2^ energy, thus making it orders of magnitude more efficient than any commercially available e-readers. The static color of an individual pixel necessitates the use of red, green and blue (RGB) pixels for a full color switching device. Similarly, Xiong et al. realized Cu plasmonic nanohole array F–P resonant cavities incorporating a PEDOT:PSS switching layer to achieve sub 1 s on-off switching times [85]. A single switching of the cavity device requires only 5.7 mJ/cm^2^, making this platform highly suitable for chromatic e-readers and plasmonic displays. In 2021 Gugole et al. published a comprehensive comparison of inorganic and organic switching materials for these kinds of static cavities [86]. Notably, their findings suggest that while organic EC switching materials, such as PEDOT:PSS and PProDOTMe_2_ allow for faster switching times (*τ*_c,b_ < 1 s) and better electrochemical cycling stability (>10^5^), other metrics, such as optical reflectance modulation and bistability are inferior when compared to WO_3_. WO_3_-based e-paper devices exhibit bistability over many hours along with stability over 100 s of electrochemical switching cycles. While both technologies have an advantage over conventional electrophoretic e-readers in terms of the achievable optical reflectance modulation and color range, they both suffer from long term instability.

Following the static examples of EC cavities introduced by Xiong et al., several resonant cavity devices with active dielectrics were investigated. First, Wang et al. pioneered WO_3_ on W cavities for color tunable display applications. Figure 5(c) shows the CIE color space of selected WO_3_ on W nanocavities as a function of the WO_3_ thickness and intercalation [39]. By increasing the thickness of the EC layer from 152 nm to 250 nm, all colors of the visible spectrum can be represented. Moreover, when redox cycling a 163 nm thick WO_3_ on W F–P resonator, the reflected color can be dynamically shifted from red to green (Figure 5(d)). When employed as supercapacitors with two WO_3_-based electrodes working in tandem, these cavity-type color displays achieved high areal capacitance of up to AC = 23 mF/cm^2^ and a high energy density of 1.13 × 10^−3^ mW h/cm^2^ at a charge current density of 0.05 mA/cm^2^ while at the same time having a high coloration of CE = 149 cm^2^/C [38]. The supercapacitor architecture retained over 92% of its capacitance when cycled 3000 times, creating the possibility for color tunable EC energy storage devices. However, chromaticity in these F–P cavities is rather low, with spectral widths routinely exceeding a full width at half maximum (FWHM) > 100 nm. The lack in chromaticity is due to the rudimentary device design, which relies solely on the resonance and refractive index dispersion of WO_3_ in the cavity. As such, solutions to improve chromaticity and spectral widths need to be found. Furthermore, the investigated WO_3_ cavities exhibit switching times of several seconds, making integration in e-readers rather difficult.

**Figure 6:**
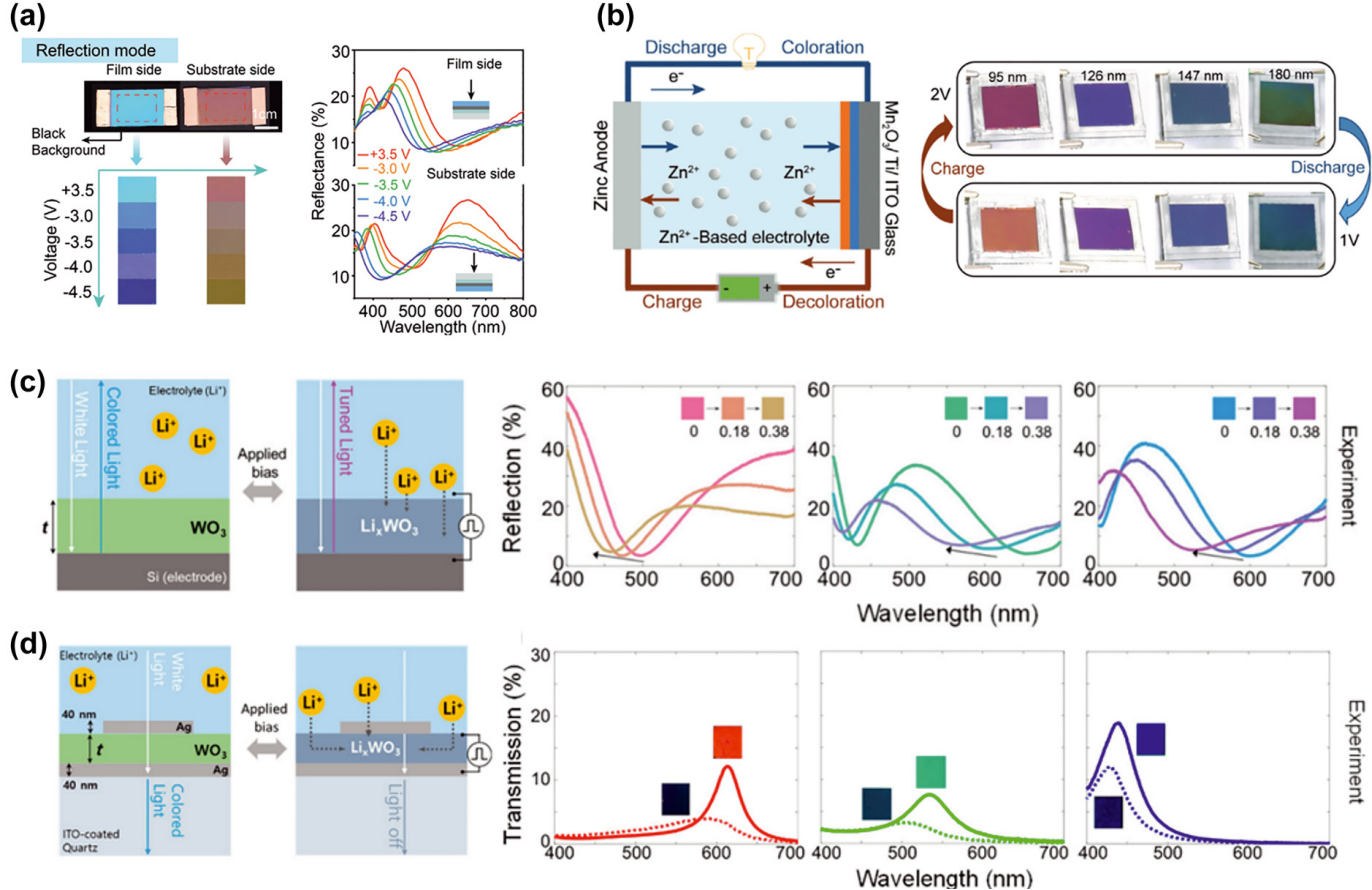
Advanced EC color displays. (a) Reflection mode of EC color display devices. Reflectance spectra measured from film and substrate sides exhibiting asymmetric colors [87]. (b) Mn_2_O_3_-based EC battery with high color tunability [88]. (c) and (d) EC cavity color devices developed by Lee et al. [80]. (c) Optical reflection-type device and corresponding optical reflectance spectra. (d) Optical transmission-type device and its corresponding optical transmission spectra [89].

Considering the simple device structure of a WO_3_ cavity and ITO on PET transparent electrodes in a PMMA-LiClO_4_ gel electrolyte, Chen et al. reported that thin (*d*_*t*_ = 4 nm–8 nm) metallic reflectors (e.g., W, Cu, Ag, Ti) led to a significant difference in the reflectance from the backside of the device as compared to the film side (Figure 6(a)) [87]. The butterfly-like reflection asymmetry is strongly dependent on the material used for the thin metallic, since higher refractive indices lead to a stronger phase shift between front and backside reflection. Similar to the work of Wang et al. the initial color of the device can be adjusted by the thickness of the WO_3_ and the ITO layers [39].

Zhao et al. presented multiple approaches for colorful EC reflective-type displays based on F–P cavities [14, 88]. In addition to the conventional cavity material, both WO_3_ and Mn_2_O_3_ were shown to exhibit superior EC properties when employed in a battery-type color device. Here, Mn_2_O_3_ is deposited onto Ti and used as the counter electrode in a zinc ion battery (ZIB) platform. Through the F–P resonance, the poor EC performance of manganese oxide is overcome, and several distinct colors can be displayed in a charge–discharge cycle (Figure 6(b)). Furthermore, the EC ZIB was shown to offer a high specific capacity of 283 mA h/g at 0.2 A/g current density and 64% charge capacity retention over 800 cycles. Ultimately, it was demonstrated here that through careful device design and material choice, rather weak EC effects in certain oxides can be enhanced through metasurfaces and cavities.

Lee et al. presented two distinct device architectures for EC cavity color displays. Their reflective device, shown in Figure 6(c), achieves up to Δ*λ* = 107 nm wavelength shift throughout the visible spectrum which is set by the WO_3_ layer thickness [80]. However, a novel approach for transmission-type devices is shown in Figure 6(d), where plasmonic Ag structures on top of a WO_3_ cavity are used to create narrowband transmission. While the plasmon interaction improves the FWHM so that highly chromatic colors are achieved, the device offers mainly static colors with a maximum wavelength shift Δ*λ* = 30 nm.

**Figure 7:**
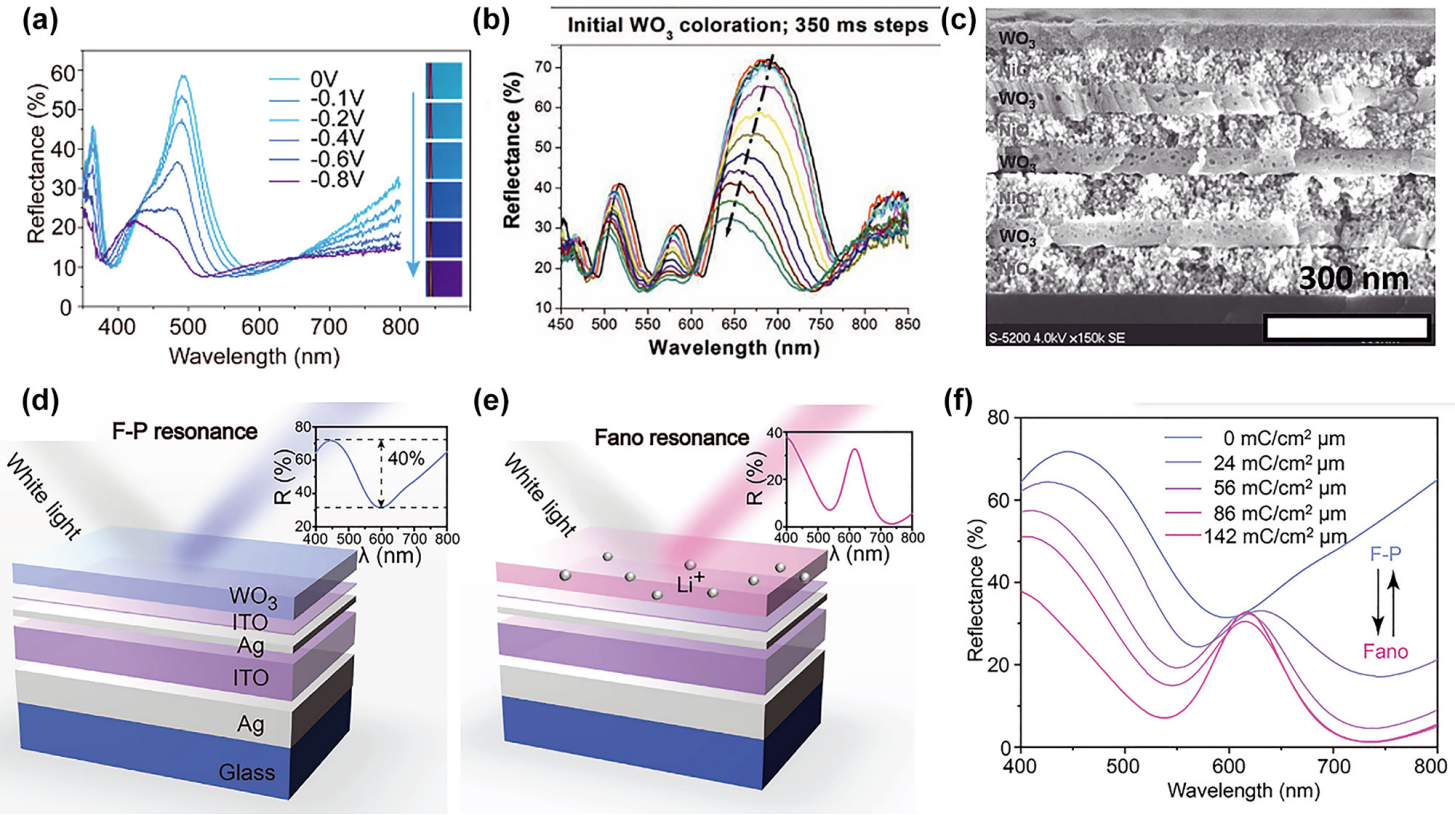
EC Bragg-Reflector-type color displays. (a) A sputter-deposited multilayer EC device exhibiting sharp resonances in the lithiation-dependent reflectance spectra [14]. (b) Reflectance spectra during coloration and (c) SEM image of device architecture [90]. (d) Schematic diagram of cavity in the F–P resonance mode. Inset: reflection spectra of the proposed structure in the F–P resonance mode. (e) The same device in Fano resonance mode (Fano reflection in inset). (f) Reflection spectra as a function of lithiation with resonance mode indicated [91].

To increase the chromaticity of reflection-type ECD displays, different approaches incorporating multilayer architectures have been introduced. By employing alternating multilayers of W/WO_3_, the initial FWHM from the Bragg-reflector can be reduced to 60 nm, leading to higher chromaticity and a 40% wider color gamut (Figure 7(b)) [14]. However, FWHM widening during coloration is also accompanied by a small change in color since only the top layer of the device can be actively modulated. In this configuration, ion permeable reflective electrodes are necessary to allow for redox reaction occurring throughout the whole device. Another type of Bragg-reflector ECD employing spun coated alternating stacks of NiO and WO_3_ nanoparticles to (Figure 7(a)) was realized by Redel et al. [90]. The consistent film thicknesses throughout four device layers, resulted in a distinct change in the reflectance spectrum when Li^+^ ions are driven into the Bragg-reflector. However, this device only achieved 25 nm total color shift, mainly owing to the arising defects and inhomogeneities from the colloidal fabrication process and the low ion intercalation of bottom layers.

Chen et al. recently improved the concept of an active EC nanocavity by means of a complex device design that allows switching between an F–P cavity resonance and a Fano resonance through the lithiation of WO_3_ (Figure 7(d) and (e)) [91]. Such a multifunctional nanophotonic platform consists of a simple F–P cavity on top of an ITO layer that sandwiched between an Ag mirror and a thin Ag reflector/absorber. In the delithiated state, the F–P cavity resonance dominates, and the device reflects a blue light color. However, when the device is lithiated, the Fano resonance dominates since the WO_3_ layer becomes highly semiconducting, making ITO the main cavity material and color changes to purple due to the mixing of the blue light color cavity resonance and the red-light color arising Fano resonance. These lithiation-dependent reflection spectra are shown in Figure 7(f) wherein during lithiation a distinct, fixed peak around 610 nm arises. Moreover, the device exhibits EC switching in *τ*_C_ = 12.2 s and *τ*_B_ = 5.6 s and when the Ag mirror is replaced with a 10 nm-thick Ag film, it exhibits different colors for transmission and reflection, respectively; as such, it is behaving as a color filter.

In another approach the organic cavity material PEDOT:PSS is patterned by UV radiation and cycled in KCl to create color modulation over an Au mirror [92]. Such a device promises easy scalability through lithographical techniques as well as fast switching speeds based on the polymer EC material. Rossi et al. further found that (poly(thieno[3,4-b]thiophene) as the electroactive cavity material achieves high tuning range of up to 365 nm from 472 nm peak position at +0.8 V to 837 nm at −1.0 V [93]. Such a high dynamic change was found to derive from a volumetric change of the material in addition to the modulation of optical properties during redox cycling. The introduced devices open an avenue for polymer-based color tuning devices. However, the device presented by Rossi degrades quickly with the peak wavelength of the reflectance decreasing from 940 nm to 820 nm over 4 cycles of redox cycling.

**Figure 8:**
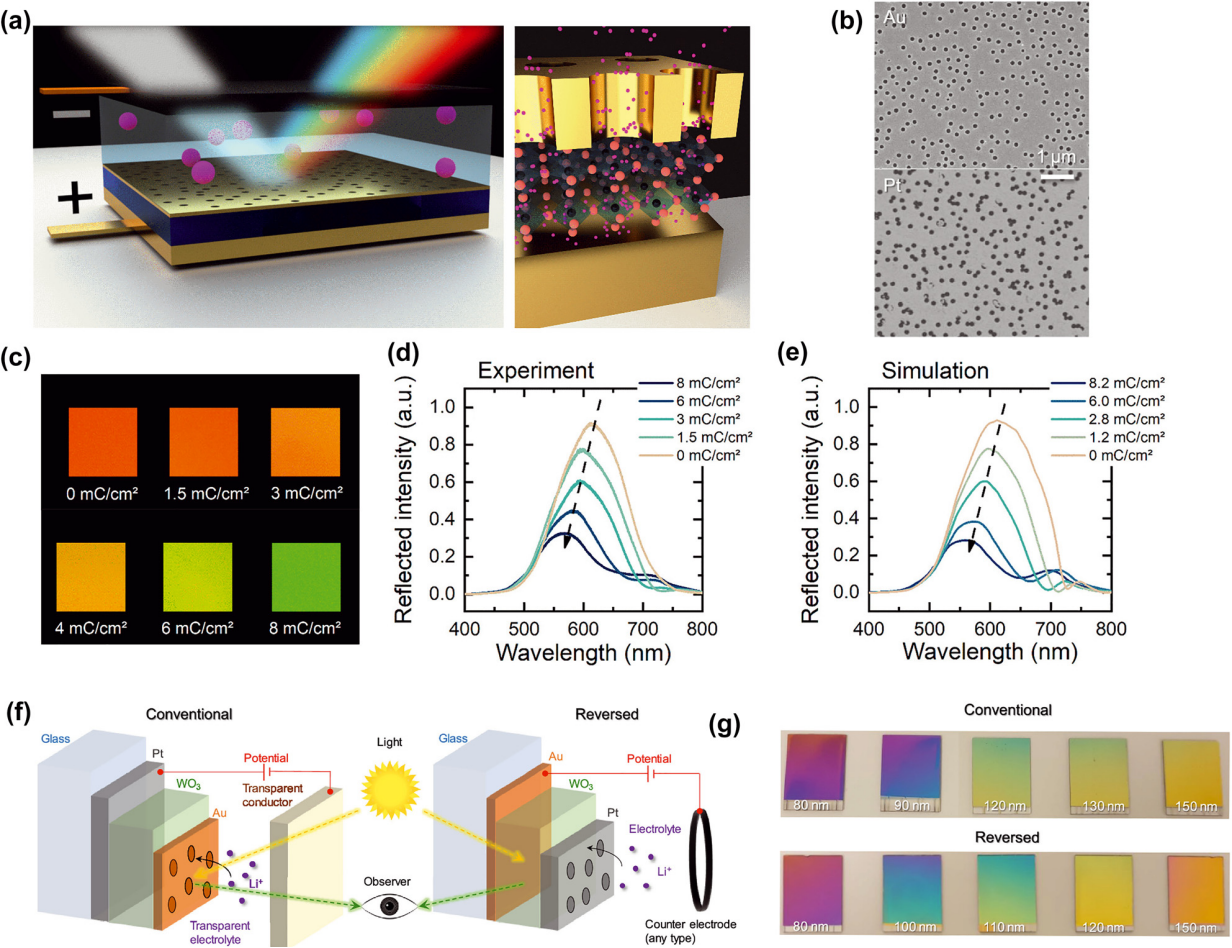
Fabry–Perot nanocavity with plasmonic NHA. (a) Schematic of a cavity-type device having NHA (Li^+^ ions in pink). (b) SEM images of random NHA fabricated in Au and Pt films. (c) Achieved colors in WO_3_ plasmochromic nanocavity for different levels of lithiation [15]. (d) and (e) Corresponding reflectance spectra as measured and simulated for a 120 nm-thick WO_3_ film. (f) Schematic of conventional and inverted device design of an F–P nanocavity with plasmonic Pt NHA. (g) Large rage colors generated by Gugole et al. [94].

To realize full color switching of a nanocavity-type device having high chromaticity, Hopmann et al. introduced an active WO_3_ nanocavity with a plasmonic NHA superstructure shown in Figure 8(a) [15]. This approach combines the static MIN cavity technology and EC switching of the cavity material for higher color clarity. Here, the WO_3_ resonator material is sandwiched between an Au mirror and an NHA electrode on the top. NHAs were fabricated through colloidal lithography (Figure 8(b)) and exhibited a random short-range order for plasmonic activity while also providing ionic pathways for intercalation [81]. The so-called plasmochromic nanocavity device, showed a significant improvement in the spectral shape of the reflectance through the overlap of the resonator mode and plasmonic extinction. Such a device provides color switching from red to green over the course of lithiation from 0 to 8 mC/cm^2^ (Figure 8(c)). It was further shown that the maximum peak shift Δ*λ* = 105 nm is very consistent between measurement and simulation (Figure 8(d) and (e)), indicating that FDTD simulations accurately describe the plasmochromic device characteristics. Gugole et al. improved this work by introducing a Pt NHA in an active EC plasmonic cavity (Figure 8(f)). By reversing the layer architecture and measuring reflection through the glass substrate rather than through the electrolyte, the reflectivity of the cavity increased by up to 15%. When this device operates between *V* = ±1.5 V, it achieves up to Δ*λ* = 110 nm spectral shift, while by adjusting the thickness of the WO_3_ layer, colors between violet and red are realized. It is important to note that a single switching cycle of the Pt NHA EC plasmonic cavity requires ∼40 mJ/cm^2^, which is below the energy density required to operate an e-ink reader.

Further development of F–P cavity-type color devices makes use of broadband absorbers in the same device architecture to enhance chromaticity of the main reflection. Ma et al. introduced a Cr-based absorber in front of the F–P cavity to shape the reflectance [95]. The combination of these two device layers does not impede on the available color shift and only reduces the reflectance by less than 5%. Other possibilities to increase switching contrast and chromaticity is the introduction of several layers of NHA into the WO_3_ active dielectric. Evidently, the NHA-based devices provide another degree of freedom that can be used to tune the optical properties of the cavity.

Thus far two distinct types of resonant cavity devices have been investigated: cavities with EC for on-off switching functionality and dynamic cavities with WO_3_ as the resonator material. While EC on-off switching based on conductive polymer coatings can be switched at video frequencies (25 Hz), their longterm stability is limited. Nonetheless, as they offer a freedom in the selection of the cavity material and the plasmonic superstructures, highly chromatic devices operating over a wide range of the visible spectrum can realized. On the other hand, EC materials as the active resonator, lead to a wide range of dynamically achievable colors; however, they lack switching speed and contrast necessary to compete with other display technologies. Notwithstanding, dynamic cavities have the potential to be integrated as indicators of the stored charge in energy storage devices. The parameters introduced for F–P-type cavities can be found in Table 2.

**Table 2:** Comparison of selected Fabry–Perot-type cavity devices. Values for color range, FWHM and contrast are extracted from figures and graphs from the corresponding references. On–Off refers to devices where the EC material is used only to absorb reflected light. Active cavities dynamically tune the reflected wavelengths.

Technology	Type	Color range (nm)	FWHM (nm)	Contrast ΔR	Ref.
Al_2_O_3_ cavity, Au NHA, EC polypyrrole	On–off	>300 (static)	70	85%	[84]
W/WO_3_ SC	Active	∼120	120	35%	[39]
Ti/Mn_2_O_3_ ZIB	Active	∼130	140	10%	[88]
Au/WO_3_/Au NHA	Active	∼105	110	70%	[15]
Ag/WO_3_/Ag nanodisks	Active	∼107 (reflection)	60	20%	[89]
		∼23 (transmission)			
W/WO_3_ multilayer	Active	∼55	55	40%	[14]

The main conclusions that can be drawn are that while both technologies provide low power consumption at operating voltage below ±2 V and are scalable for large-scale production, a major drawback to overcome is that all investigated devices to date used liquid electrolytes, which limits their potential for integration into e-readers or similar display platforms. With switching speeds as high 25 Hz for EC on-off switching with polymer EC materials of resonant cavity devices and full color modulation in less than 3 s with WO_3_ [15, 30, 85], such a platform shows tremendous promise in replacing common e-readers based on e-ink and OLED displays wherever switching speeds are less important than energy consumption. While OLED displays require a power density of roughly 15 mW/cm^2^ and e-readers about 3 mW/cm^2^, plasmonic cavity devices have shown to be a lot more energy efficient (*P* < 1 mW/cm^2^) [84]. However, full color modulation is necessary to even compete with LED displays.

### 2.4 Dielectric nanophotonic electrochromic devices

#### 2.4.1 EC optical modulation materials for integrated photonics

From the review above, it is evident that F–P nanocavities are promising platforms for nanophotonic ECDs. To present a full picture of the past and future trends of ECDs, it is worth discussing the use of EC materials for advanced EC optical transmission modulation in waveguide-type devices. While the modulation of resonant effects is mainly reliant on the change in the refractive index of the EC material, the inherent increase of optical extinction can be utilized in opical transmission modulation for photonic devices such as optical waveguides. To date, EC modulation of optical waveguide transmission is realized in devices based on the conventional “smart window” architecture where the electrical contact in direct proximity to the waveguide core [40, 41, 96].

The first report of EC waveguide fabrication and measurements was by Page et al. in 1990 [97]. However, the authors rather focused on utilizing the EC effect to demonstrate optical poling of an electro–optic polymer exhibiting EC properties. However, the dependence on the EC induced optical poling and the light absorption inspired further investigations of such a platform. In 1992, Piraud et al. presented the first EC waveguide modulator based on a planar optical waveguide [98]. Their waveguide platform was based on the EC effect in lutetium biphthalocyanine (Lu(PC)_2_). The device consisted of planar potassium-exchanged sodalime glass waveguide with a silica buffer layer between the core material and the ITO electrode on which the Lu(PC)_2_ film is deposited. The resulting device having a 10 nm thick Lu(PC)_2_ film and 200 nm buffer thickness between waveguide core and 10 nm ITO film exhibits modulation of the TE mode optical absorption along the waveguide from 10 dB/cm to 22.5 dB/cm at *λ* = 680 nm. It was further found that the thickness of the spacer layer, as well as the thickness of the EC material, have a strong influence on the achievable modulation and on-state transmission of the device. Since, the EC effect in Lu(PC)_2_ is triggered by Cl^−^ ions, this particular device was envisioned as an integrated EC sensor. The novelty of such an EC platform is the decoupling of ionic diffusion path length and light propagation direction. While conventional ECDs necessitate a trade-off between switching speed and contrast due to the thickness of the EC layer, the present EC waveguide platform offers a design versatility for optimal functionality.

**Figure 9:**
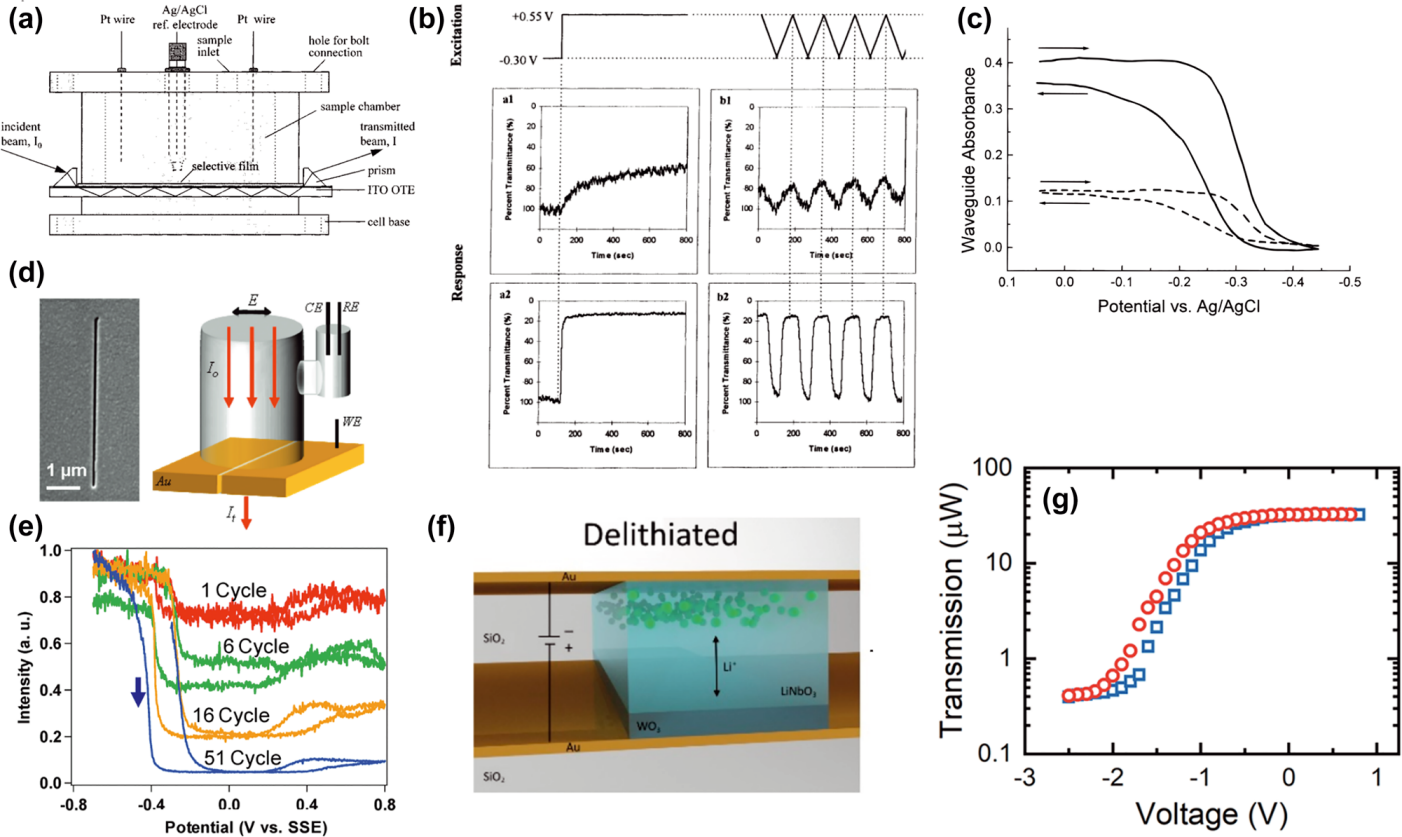
EC waveguide attenuation technology. (a) Integrated optical waveguide cell as introduced by Shi et al. with (b) electrochromic response for 0.025 mM (top) and 2.5 mM (bottom) PB concentration [100]. (c) Voltage-dependent waveguide attenuation for 0.5 µM and 2 μM PB concentration [99]. (d) SEM image of nanoplasmonic switch and a schematic of measurement cell. (e) Transmittance of nanoplasmonic switch as a function of voltage and cycle number [96]. (f) Schematic illustration of a solid-state plasmochromic waveguide and (g) its corresponding voltage-dependent transmission [42, 96, 99, 100].

The development of EC optical waveguide has found niche applications in sensing and detection of sub-monolayer adsorbate coverage. Dunphy et al. were able to detect less than 1% of a monolayer surface coverage of K_4_Fe(CN)_6_ (Prussian Blue, PB), in an integrated optical waveguide [99]. During redox cycling of PB, the absorbance in the waveguide is strongly correlated to the surface coverage of the EC material, as well as electrolyte concentration. In a similar approach, Shi et al. reported a detection limit of as low as 8 × 10^−6^ M for PB [100]. Such devices make use of the strong interaction between optical mode propagating along the waveguide and the EC material to directly correlate the optical transmittance to analyte concentration. A schematic of a typical measurement cell is shown in Figure 9(a) where the propagating wave is in direct contact to the analyte film and contained in an electrochemical cell. Depending on the concentration of PB in the analyte solution, optical transmission can be modulated by 40%–85% in less than 5 s (Figure 9(b)) [100]. Similarly, Dunphy et al. showed that the optical absorbance during CV cycling in different concentrations of PB (Figure 9(c)).

The utilization of EC modulation for integrated optics and photonics was later demonstrated in form of an electrochromic variable optical attenuator (ECVOA) by O’Brien et al. [101]. In this low insertion loss (0.5 dB) NIR spectral optical modulator, an all-solid-state WO_3_ ECD with Ta_2_O_5_ as the proton conductor is utilized to modulate the attenuation of a gradient index lens by up to 12 dB. Remarkably, owing to the fast proton conduction, the attenuator exhibits up to 5 dB/cm switching rate and high stability over 70,000 switching cycles [92]. However, EC switching in photonic devices was not further pursued until Agrawal et al. demonstrated ∼96% optical transmission modulation of PB (deposited from 5 mM K_4_Fe(CN)_6_ in 0.1 M HClO_4_) in a 50 nm × 5 µm nanoplasmonic slit waveguide [96] (Figure 9(d)). In comparison to a flat gold surface with electrodeposited PB films, the nanoslit device achieves roughly 60% higher optical contrast of up to 96% (Figure 9(e)). Thus, the nanoslit experiment hints towards superior ECDs with orthogonal paths for ion diffusion and light propagation.

In another plasmonic waveguide approach, Hopmann et al. made use of the intrinsic field localization due to plasmonic coupling and introduced a nonresonant plasmonic waveguide optical modulator based on a multifunctional solid-state LiNbO_3_ [42]. In this platform, the ionic conductor (LiNbO_3_) not only allows for plasmon propagation along a metal-insulator-metal (MIM) waveguide, but it also provides excess Li^+^ ion conduction and subsequent redox cycling of a 50 nm thin WO_3_ layer (Figure 9(f)). For a 10 µm long plasmonic waveguide, the CV-dependent transmission decreases from 32 µW to roughly 0.4 µW (Figure 9(g)). Such a device exhibits 38 dB optical transmission modulation along a 20 µm waveguide. Through decoupling of the plasmon propagation and ion diffusion paths, the EC contrast is hence increased by a true solid-state device more than three orders of magnitude while reducing the EC switching times to *τ*_EC_ < 5 s for. The solid-state nature of the ionic conduction layer further leads to extremely high electrochemical stability for over 200 redox cycles and retention of 97% of the optical transmittance modulation.

**Figure 10:**
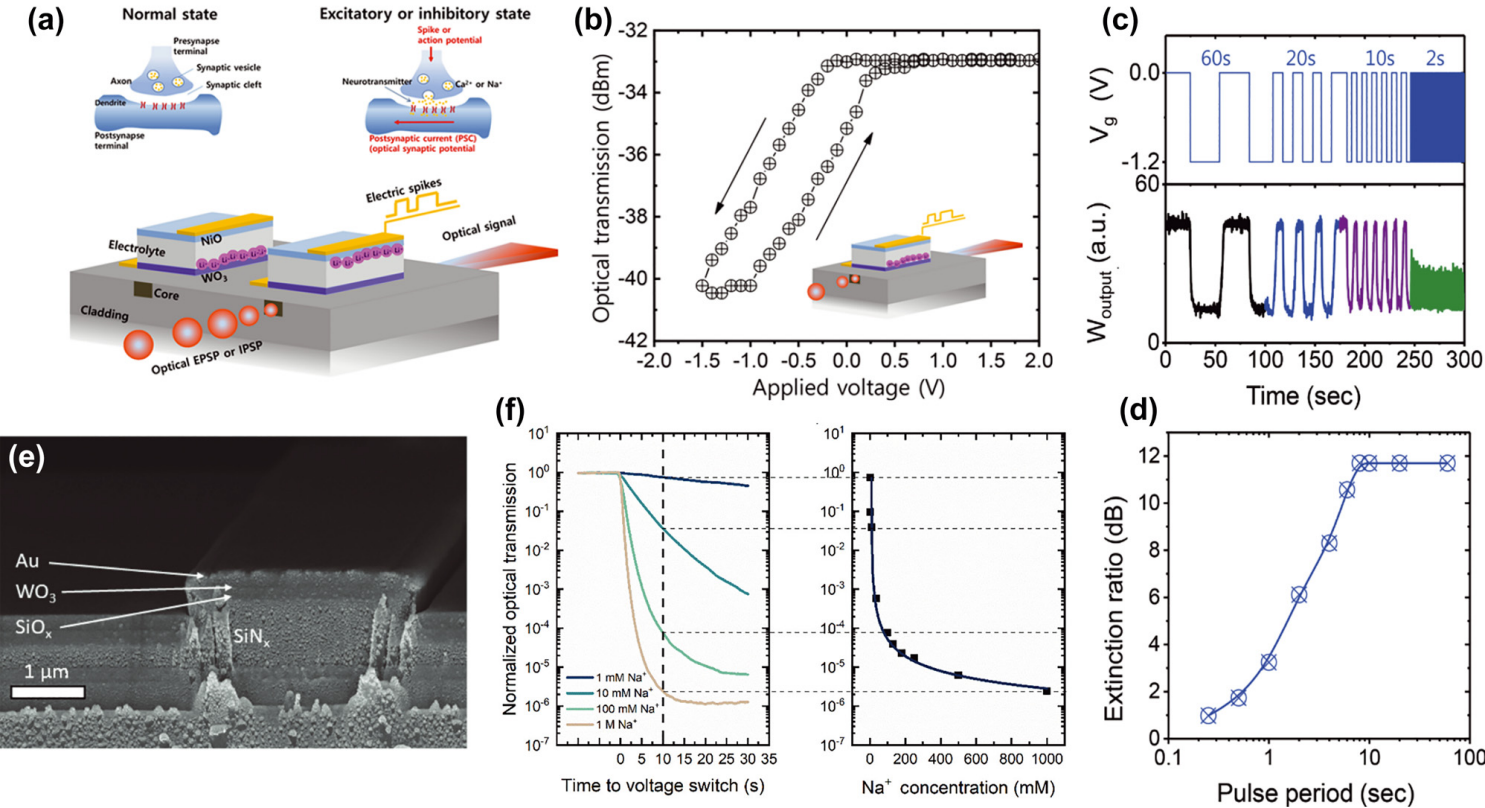
Nanophotonic EC waveguide technologies. (a) Schematic of synaptic transmission modulation via Ca^2+^ ions and corresponding device design. (b) Voltage-dependent optical transmission in the artificial synapse [40, 41]. (c) Duty cycle dependence of the electrochromically modulated waveguide device with (d) optical extinction to pulse period ratio. (e) FESEM image of Silicon Nitride-based Na^+^-ion sensor. (f) Optical transmittance as a function of time and Na^+^ concentration with calibration curve for ion sensing [107].

Kim et al. introduced optically readable EC waveguide modulator devices acting as artificial synapses for ionically driven bio-applications (Figure 10(a) and (b)) [40, 41]. Ionic gating of the EC materials has shown promise for bistable resistive switching devices and on-chip memory. However, the utilization of the optical signal modulation is lacking in these devices [102–106]. To incorporate EC-based optical modulation at increased speed and optical transmission modulation, a conventional smart window-type ECD is built on top of a buried polymer waveguide with cladding. The WO_3_ layer on top of the waveguide core modulates the optical signal traversing through the waveguide, effectively increasing the light–matter interaction with the EC oxide to the length of the waveguide. The devices introduced here achieved 10 dB optical switching modulation with sub-second switching speeds, which is up to 5 times faster than conventional EC cycling of WO_3_. However, the initial loss due to the coupling of the optical mode to the electrical contact necessary to drive ions into the EC material are rather large and prevent these devices from reaching the full potential of EC transmission modulation (Figure 10(c) and (d)).

By means of device design, recently, Hopmann et al. offered an interesting solution to overcome the low optical transmission associated with mode metal electrodes coupling in EC-based optical waveguides (Figure 10(e) and (f)) [107]. Here, an EC WO_3_ layer is sputtered onto a Silicon Nitride waveguide cladded by Silicon Oxide. The electrical contacts are placed several micrometers off to the side of the waveguide core to prevent coupling of the optical mode. Such a design makes use of the inherent electrical and ionic conductivity of WO_3_, where the voltage drop over the electrode-free distance is negligible when compared to the voltage drop over the electrolyte. The EC waveguide platform exhibits up to 70 dB transmission modulation with strong dependence on the Na^+^ ion concentration in the electrolyte. As such, the nanophotonic waveguide can be employed as a Na^+^ sensor with accuracies >90% in the concentration range from 1 mM to 1 M. The platform further provides the EC switching speed necessary to facilitate real-time measurements (*τ*_EC_ < 0.56 s).

A major challenge for EC waveguide technology is the miniaturization to the nanoscale, with all devices published to date relying on liquid electrolytes and device geometries on the scale of several millimeter. To achieve full, on-chip transmission modulation for EC waveguides, truly nanoscale device design and all-solid-state approaches are essential. Furthermore, introducing enhanced light–matter interaction through plasmonic structures can help increasing the inherent absorption of the EC material [108]. On the other hand, the waveguide devices introduced to date show great promise for ion-specific real-time microfluidic sensing. Most EC materials show specific selectivity, such as Cl^−^ for Lu(PC)_2_, OH^−^ for PB or Li^+^, Na^+^, and Al^3+^ for WO_3_. Planar devices made from WO_3_ have shown promise as sensors for cystic fibrosis or as gas sensors [17, 19]. As such, EC waveguides would help increase sensitivity and contrast for such devices. In such devices, operation in transmission or multiple reflections artificially increase the light–EC interaction volume to enhance the absorption effect. The need for nanoscale fabrication necessitates materials that are compatible with standard micromachining processes, which favors inorganic materials.

## 3 Summary and outlook

Nanoscale ECDs having advanced functionalities based on the exploitation of photonic and plasmonic resonances have opened new frontiers for EC research. Despite the limitations of the EC materials, many of the shortcomings, such as static colors, slow switching speeds, and limited optical contrast are being overcome through nanoscale integration and incorporation of nanoplasmoic structures and resonant cavities. While proof-of-principle devices have been introduced decades ago, modern nanofabrication technology is helping to bring the promise of these devices to fruition. For example, the first EC modulation of plasmonic resonances of Ag nanoparticles dates to back to 2003 where it achieved 60 nm wavelength shift; however, more recently, the direct growth of PANI on Au nanospheres led to the development of full color, fast switching EC pixels [30]. Other examples of the advances made in recent years are full color EC F–P cavities that span the whole visible spectrum.

The broad impact of the present ECD research is expected to come from the integration of resonant nanoplasmonic cavities and Fano resonant structures with solid-state EC materials to alter the wavelength selectivity range, improve color chromaticity, and enhance both the optical transmissive and reflective properties of the device. The ECD technologies developed in recent years are merely building the foundation for advanced devices having functionality in display applications, medical, and environmental sensing and even telecommunication. One can envision plasmochromic reconfigurable displays based on targeted deposition and redeposition of plasmonic particles. The potential for EC photonics is endless, with devices already proving that such a technology can achieve fast switching speed and the modulation depth for high sensitivity ion detection.

For ECD color change devices, it is imperative that solutions are found to include the whole visible spectral range. Such a change is difficult to achieve either due to the limited range of plasmonic resonances in nanostructures, which usually span only part of the VIS spectrum and on the other hand the refractive index change in WO_3_ is not high enough to create an FP nanocavity dynamically tuning over the whole range. Hence, the solution here is either multipixel devices such as the ones used in RGB LEDs or nanoengineering of the involved materials to create wider resonance ranges.

In future, more research is needed to find highly stable solid-state electrolytes with sufficient switching speed. A future for ECDs in displays and integrated photonics is only feasible if modulation speeds are comparable to existing technologies. Hence, for colorful e-reader applications, long lifetime solid-state ECDs with switching speeds of several milliseconds have to be realized. Another example is the direct monitoring of salt/electrolyte concentrations with EC waveguide technology, which can only be assumed to compete with conventional sensors if live measurements are possible.

While there are several key issues, such as switching speed, life time, color range, and necessity of solid-state electrolytes, that have to be addressed when exploring a future for ECD innovations, the inherent prospect of a low power consumption and bistable nanophotonic device based on EC materials warrants the effort put into its research.
